# The Receptors that Mediate the Direct Lethality of Anthrax Toxin 

**DOI:** 10.3390/toxins5010001

**Published:** 2012-12-27

**Authors:** Shihui Liu, Yi Zhang, Benjamin Hoover, Stephen H. Leppla

**Affiliations:** Laboratory of Parasitic Diseases, Microbial Pathogenesis Section, National Institute of Allergy and Infectious Diseases, National Institutes of Health, Bethesda, MD 20892, USA; E-Mails: zhangy23@niaid.nih.gov (Y.Z.); hooverbj@niaid.nih.gov (B.H.)

**Keywords:** anthrax, CMG2, FP59, integrin β1, Tem8

## Abstract

Tumor endothelium marker-8 (TEM8) and capillary morphogenesis protein-2 (CMG2) are the two well-characterized anthrax toxin receptors, each containing a von Willebrand factor A (vWA) domain responsible for anthrax protective antigen (PA) binding. Recently, a cell-based analysis was used to implicate another vWA domain-containing protein, integrin β1 as a third anthrax toxin receptor. To explore whether proteins other than TEM8 and CMG2 function as anthrax toxin receptors *in vivo*, we challenged mice lacking TEM8 and/or CMG2. Specifically, we used as an effector protein the fusion protein FP59, a fusion between the PA-binding domain of anthrax lethal factor (LF) and the catalytic domain of *Pseudomonas aeruginosa* exotoxin A. FP59 is at least 50-fold more potent than LF in the presence of PA, with 2 μg PA + 2 μg FP59 being sufficient to kill a mouse. While *TEM8^−/−^* and wild type control mice succumbed to a 5 μg PA + 5 μg FP59 challenge, *CMG2^−/−^* mice were completely resistant to this dose, confirming that CMG2 is the major anthrax toxin receptor *in vivo*. To detect whether any toxic effects are mediated by TEM8 or other putative receptors such as integrin β1, *CMG2*^−/−^/*TEM8*^−/−^ mice were challenged with as many as five doses of 50 μg PA + 50 μg FP59. Strikingly, the *CMG2*^−/−^/*TEM8*^−/−^ mice were completely resistant to the 5-dose challenge. These results strongly suggest that TEM8 is the only minor anthrax toxin receptor mediating direct lethality *in vivo* and that other proteins implicated as receptors do not play this role.

## 1. Introduction

*Bacillus anthracis* is gram-positive, spore-forming, rod-like bacterium that causes anthrax disease through bacteremia as well as by secreting exotoxins—anthrax lethal toxin and edema toxin [1]. Anthrax toxins are AB-type toxins, consisting of the cellular receptor binding moiety, protective antigen (PA), and the two enzymatic moieties, lethal factor (LF) and edema factor (EF). Each part is individually non-toxic [2]. To intoxicate target cells, PA binds to cell surface receptors. The two well-studied receptors are tumor endothelium marker-8 (TEM8, or anthrax toxin receptor 1) and capillary morphogenesis protein-2 (CMG2, or anthrax toxin receptor 2) [3,4,5]. Recent studies have reported that integrin β1 can also serve as a receptor [6]. It is also possible that other cell surface proteins may act as co-receptors by interacting with the known receptors to alter their properties or promote toxin internalization. Upon binding, PA is cleaved by cell surface furin or furin-like proteases, converting it to the active cell surface bound PA63, which then spontaneously oligomerizes to form PA63 heptamer or octamer prepores. The oligomerized PA63 has the ability to bind 3 or 4 EF and/or LF moieties, resulting in the final assembly of PA/EF or PA/LF toxin complexes on the cell surface [7]. Oligomerization of PA63 also triggers endocytosis of the toxin complex [8,9]. In endosomes, the acidic environment triggers PA63 prepore conversion to an LF- and EF-conducting channel, through which LF and EF translocate into the cytosol to exert their cytotoxic effects. 

EF, which forms edema toxin (ET) with PA, is a calmodulin- and Ca^2+^-dependent adenylate cyclase that elevates intracellular cAMP levels by converting ATP to cAMP, causing cAMP-induced cellular effects including skin edema and lethality in experimental animals [10,11]. LF, which forms lethal toxin (LT) with PA, is a Zn^2+^-dependent metalloproteinase that cleaves and inactivates the mitogen-activated protein kinase kinases [12,13,14], affecting many cellular functions that depend on the ERK, p38, and JNK mitogen-activated protein kinase (MAPK) signaling pathways. LT in sufficient doses can lead to host death [15]. 

The receptors TEM8 and CMG2 contain a signal peptide, an extracellular von Willebrand factor A (vWA) domain, a single-pass transmembrane region (TM) for plasma membrane anchoring, and a cytosolic tail that might be involved in cytoskeleton interaction and is subject to certain post-translational modifications [16,17]. CMG2 and TEM8 share 60% sequence identity in their vWA domains, which contain a typical metal ion-dependent adhesion site (MIDAS) motif responsible for PA binding [17]. We have previously generated TEM8- and CMG2-null mice (*CMG2^−/−^* and *TEM8^−/−^*, respectively) by deleting their TM domains. Through analyzing these mice, we have shown previously that CMG2 is the major anthrax toxin receptor *in vivo* and TEM8 only plays a minor role in anthrax toxin pathogenesis [5]. This is due, at least partially, to the 10-fold higher affinity of PA for CMG2 as compared to TEM8. Recently, another cell surface vWA domain-containing protein, integrin β1, was reported to mediate killing of the mouse macrophage cell line RAW264.7 by LT, suggesting that integrin β1 is another anthrax toxin receptor [6]. The integrin α4β1 and α5β1 complexes were reported to have a PA-binding affinity similar to TEM8 [6]. Whether integrin β1 complexes or other vWA domain-containing proteins functioning as anthrax toxin receptors *in vivo* remains as an important issue to be addressed.

## 2. Results and Discussion

**Figure 1:**
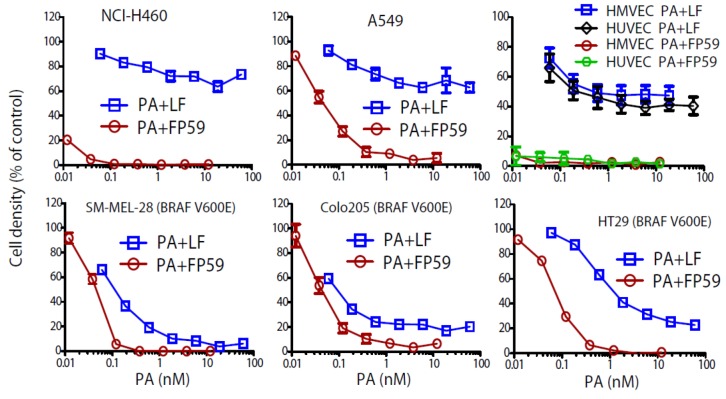
PA + FP59 is much more toxic than LT (PA + LF) to cells. Sensitivities of NCI-H460 cells, A549 cells, human umbilical vascular endothelial cells (HUVEC), human microvascular endothelial cells (HMVEC), SM-MEK-28 cells, Colo205 cells, and HT29 cells to PA/FP59 and LT are shown. Cells cultured in 96-well plates were treated with various concentrations of PA (0–12 nM) + 1.9 nM FP59 or various concentrations of PA (0–60 nM) + 6 nM LF for 48 h. Cell viability was evaluated by MTT assay. Untreated cells were used as a reference to calculate percent survival. The bottom three panels are the cells with the BRAF V600E mutation. Data are reported as mean viability ± S.D.

Although LT and ET can, acting as the major virulence factors of *B. anthracis,* induce lethality in experimental animals, neither toxin affects the viabilities of most cell types [1]. So far, ET has not been found to be able to kill any cell type or cell line. Although macrophages that contain LT-sensitive Nlrp1b can be killed by LT through pyroptosis [18,19], proliferation of most other cell-types can only be modestly affected by LT. Recently, human tumor cells having the BRAF V600E mutation were found to be sensitive to LT [20,21]. These cells have developed a reliance on the MEK-ERK signaling pathway for survival and thus can be induced to undergo apoptosis by LT. In addition to delivering the native effectors LF and EF, PA has also been used to deliver other polypeptides fused with LF amino acids 1–254 (LFn), the PA-binding domain of LF. FP59 is such a protein, in which LFn is fused to the catalytic domain of *Pseudomonas aeruginosa* exotoxin A [22]. FP59 kills cells by ADP-ribosylating the unique diphthamide residue on eukaryotic elongation factor-2 (eEF2), thereby inactivating the factor’s activity in protein synthesis [23]. Because a few FP59 molecules are enough to inactivate all eEF2 in a cell, leading to cell death, PA + FP59 is much more potent than LT to most cells. Examples of the results of cytotoxicity experiments are shown in Figure 1. PA + FP59 is much more toxic than LT to tumor cell lines NCI-H460 and A549 and primary human endothelial cells (Figure 1). Even the few LT-sensitive human tumor cells having the BRAF V600E mutation, such as melanoma SK-MEL-28, HT29 and colon carcinoma Colo205, are less sensitive to LT than to PA/FP59 (Figure 1).

**Figure 2:**
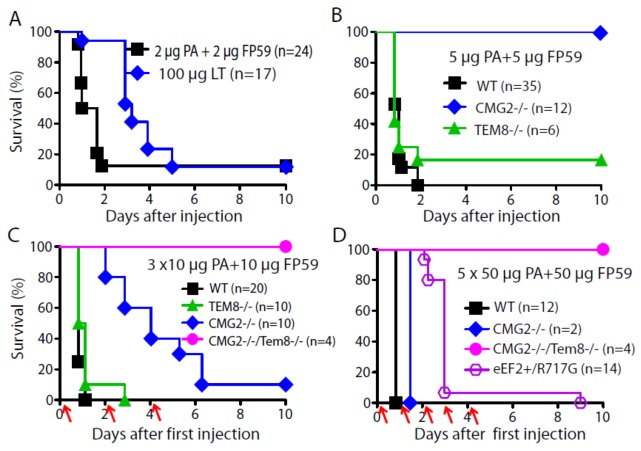
CMG2 and TEM8 are the only two anthrax toxin receptors mediating lethality *in vivo*. (**A**) WT C57BL/6 mice were challenged with either 2 μg PA + 2 μg FP59 or 100 μg LT (100 μg PA + 100 μg LF) intraperitoneally and monitored for survival; (**B**–**D**) Mice with different genotypes were challenged with either 5 μg PA + 5 μg FP59 (**B**), 3 doses of 10 μg PA + 10 μg FP59 (every other day, indicated by arrows) (**C**), or 5 doses of 50 μg PA + 50 μg FP59 (every day, indicated by arrows) (**D**) and monitored for survival.

We then compared the toxicity of PA + FP59 and LT in mice. We found that PA + FP59 was also much more toxic than LT to mice; only 2 μg PA + 2 μg FP59 killed 90% of the mice (Figure 2A), a degree of lethality reached only with 100 μg of LT (100 μg PA + 100 μg), showing that PA + FP59 is at least 50-fold more potent than LT *in vivo*. Thus, the *in vivo* toxicity of FP59 mediated by PA can provide a more sensitive indicator for detecting toxic effects mediated by putative additional receptors such as integrin β1 [6]. To ascertain whether CMG2 plays a dominant role in mediating the *in vivo* toxicity of PA + FP59, the *CMG2^−/−^*, *TEM8^−/−^*, and WT control mice were challenged with 5 μg PA + 5 μg FP59. While all the WT controls (35/35) and 80% of *TEM8^−/−^* mice (5/6) succumbed to the challenge, all of the *CMG2^−/−^* mice survived (Figure 2B), confirming that CMG2 is the major anthrax toxin receptor *in vivo*. These results showed that the amounts of PA + FP59 internalized by TEM8 and potentially integrin β1 were insufficient to induce lethality when 5 μg PA + 5 μg FP59 was used. In order to detect any toxic effects mediated by TEM8 and/or other potential minor receptors, the *CMG2^−/−^* and *CMG2^−/−^*/*TEM8^−/−^* (double knockouts) mice were challenged with 3 doses of 10 μg PA + 10 μg FP59. While all the WT and *TEM8^−/−^* mice were killed (mostly within 24 h after the first injection), 90% of the *CMG2^−/−^* mice also succumbed to the challenge at a slower rate, revealing of the lethal effects induced by TEM8 and potentially integrin β1 (Figure 2C). However, all the *CMG2^−/−^*/*TEM8^−/−^* mice survived the challenge without displaying any sign of disease (Figure 2C), indicating the delayed lethal effects shown above in *CMG2^−/−^* mice were mediated by TEM8 but not integrin β1 or other potential low affinity toxin receptors. To explore whether the potential minor role of integrin β1 in mediating the toxin internalization can be detected at even higher doses of the toxin, we heavily challenged the *CMG2^−/−^*/*TEM8^−/−^* mice with 5 doses of 50 μg PA + 50 μg FP59. In this experiment we also included the *eEF2^+/G717R^* mice in which 50% of eEF2 does not have the diphthamide modification due to the G717R mutation, rendering the mice highly resistant to PA + FP59 [24]. We found that all these *eEF2^+/G717R^* mice succumbed to the 5 doses of 50 μg PA + FP59 challenge (Figure 2D). Remarkably, the *CMG2^−/−^*/*TEM8^−/−^* mice were completely resistant to the heavy challenge, not showing any sign of disease. Interestingly, after the challenge the male *CMG2^−/−^*/*TEM8^−/−^* mice continued to be successful breeders (data not shown). These results demonstrate that integrin β1 or other potential PA binding proteins are unable to mediate lethality caused by anthrax toxins *in vivo*. 

In summary, we provide evidence that integrin β1 or other potential PA binding proteins do not appear to function as anthrax toxin receptors that mediate direct lethality *in vivo*. To detect toxic effects caused by the proposed weak anthrax toxin receptor integrin β1, we used FP59 as the PA effector protein, in part because it is at least 50-fold more toxic than LF *in vivo*. PA + FP59 is extremely toxic, with 2 μg each being sufficient to kill a mouse. Intriguingly, the *CMG2^−/−^*/*TEM8^−/−^* mice were completely resistant to as many as 5 doses of 50 μg PA + 50 μg FP59 challenge, not displaying any sign of disease. These results, combined with the previous results showing that the *CMG2^−/−^*/*TEM8^−/−^* mice are completely resistant to 5 doses of 100 μg LT challenge [5], strongly suggest that integrin β1 and other potential PA binding proteins do not serve as anthrax toxin receptors mediating direct lethality *in vivo*. Therefore, CMG2 is the major anthrax toxin receptor and TEM8 is the only minor receptor mediating direct lethality *in vivo*. We cannot exclude the possibility that the potential alternative functional receptors could be present on a subset of cells whose killing by PA + FP59 is not lethal to mice. Although we have shown previously that CMG2 also functions as the major receptor at the early stage of anthrax infection [25], the role of the potential alternative receptors in anthrax infection pathogenesis cannot be excluded.

## 3. Experimental Section

### 3.1. Cell Culture and Cytotoxicity Assay

Human umbilical vascular endothelial cells (HUVEC) and human microvascular endothelial cells (HMVEC) were obtained from Cambrex (Walkersville, MD, USA). HUVEC and HMVEC were cultured in endothelial cell growth medium-2 (EGM-2) plus EGM-2 singleQuots and EGM-2 plus EGM-2 MV singleQuots (Cambrex, Walkersville, MD, USA), respectively. All tumor cells were cultured in DMEM with 10% FBS. For cytotoxicity assays, cells were grown in 96-well plates and treated with serial dilutions of PA combined with 1.9 nM FP59 or 6 nM LF for 48 h. Cell viability (density) was then assayed by MTT (3-[4,5-dimethylthiazol-2-yl]-2,5-diphenyltetrazolium bromide) as described previously [9]. 

### 3.2. Toxin Challenge Studies

PA, LF, and FP59 were purified as previously described [17,26,27]. In toxin challenge experiments, 8–10 week old male and female mice with various genotypes were injected with various doses of PA + FP59 as indicated in Figure 2, and survival monitored. 

Generation of TEM8- and CMG2-null mice and *eEF2^+/G717R^* mice were described previously [5,24]. All animal studies were carried out in accordance with protocols approved by the National Institute of Allergy and Infectious Diseases Animal Care and Use Committee. 

## 4. Conclusions

We conclude that CMG2 is the major anthrax toxin receptor and TEM8 is the only minor receptor mediating direct lethality *in vivo*, and that other potential PA binding proteins do not play this role. This study indicates that therapeutics aimed at TEM8 and CMG2 would be sufficient to block the toxins lethal actions.
